# Comparative Analysis of Three Different Methods for Monitoring the Use of Green Bridges by Wildlife

**DOI:** 10.1371/journal.pone.0106194

**Published:** 2014-08-29

**Authors:** Goran Gužvica, Ivana Bošnjak, Ana Bielen, Danijel Babić, Biserka Radanović-Gužvica, Lidija Šver

**Affiliations:** 1 Department for Biomonitoring and Nature Protection, Oikon Ltd., Institute for Applied Ecology, Zagreb, Croatia; 2 Laboratory for Biology and Microbial Genetics, Department of Biochemical Engineering, Faculty of Food Technology and Biotechnology, Zagreb, Croatia; 3 Byte Lab, Zagreb, Croatia; 4 Croatian Natural History Museum, Zagreb, Croatia; Smithsonian Conservation Biology Institute, United States of America

## Abstract

Green bridges are used to decrease highly negative impact of roads/highways on wildlife populations and their effectiveness is evaluated by various monitoring methods. Based on the 3-year monitoring of four Croatian green bridges, we compared the effectiveness of three indirect monitoring methods: track-pads, camera traps and active infrared (IR) trail monitoring system. The ability of the methods to detect different species and to give good estimation of number of animal crossings was analyzed. The accuracy of species detection by track-pad method was influenced by granulometric composition of track-pad material, with the best results obtained with higher percentage of silt and clay. We compared the species composition determined by track-pad and camera trap methods and found that monitoring by tracks underestimated the ratio of small canids, while camera traps underestimated the ratio of roe deer. Regarding total number of recorder events, active IR detectors recorded from 11 to 19 times more events then camera traps and app. 80% of them were not caused by animal crossings. Camera trap method underestimated the real number of total events. Therefore, an algorithm for filtration of the IR dataset was developed for approximation of the real number of crossings. Presented results are valuable for future monitoring of wildlife crossings in Croatia and elsewhere, since advantages and disadvantages of used monitoring methods are shown. In conclusion, different methods should be chosen/combined depending on the aims of the particular monitoring study.

## Introduction

High number of linear transport routes, especially motorways, represent one of the most severe modifications of the natural landscape today [1], [2]. The roads exhibit numerous negative impacts on wildlife populations, from habitat loss and fragmentation, barriers to animal movement and gene flow, to traffic noise, light pollution and wildlife mortality caused by animal-vehicle collisions [3]–[6]. Wildlife crossing structures are above-grade (wildlife overpasses) or below-grade (wildlife underpasses) structures designed to facilitate movement of animals, connect populations and reduce wildlife mortality. Wildlife overpasses are bridge-like structures of whatever size, designed for use by fauna or, at the most, for dual use by farm vehicles and wildlife, and planted with vegetation [6]. Besides their primary function, they may serve as an excellent monitoring place for the estimation of the population size and the ecological impact of the highway traffic on certain large animals such as brown bear [7]. Wildlife crossing design types include green bridges, wildlife overpasses, multi-use overpasses and canopy crossings, depending on the size and targeted wildlife species groups and taxa. Green bridges (also called landscape bridges) are the largest wildlife crossing structures (minimum width 70 m), designed exclusively for wildlife use. Large size enables the restoration of adjacent habitats and facilitate use by largest number of species [4].

Animal activity on the wildlife crossings can be monitored using various methods such genetic sampling (as hair/DNA snagging devices), radio and satellite telemetry tracking, road-kill or vehicle collision data, snow tracking, tracking beds, tracking plates, digital camera and video monitoring, active and passive infrared (IR) tracking systems [8]–[15]. Out of these, track-pads, digital camera traps and infrared (IR) trail monitoring systems are indirect methods especially suitable for monitoring of animal activity on the wildlife crossings [16]. Each monitoring method has advantages and disadvantages in terms of quality and nature of obtained information, as well as cost. Cameras provide proof of species presence in an area; can teach what prints and scats go with which species; for some species allow photo-identification of individuals; estimate the abundance, density and relative abundance of animal populations; allow biodiversity estimation and are a cost effective long-term monitoring tool [10], [17], [18]. Tracking is another monitoring method, where a track pad is positioned on the bridge and tracks (and scat) are periodically determined [10]. Finally, IR trail monitoring system detects an animal (or any other moving object, people etc.) when it blocks or reduces an IR signal transmitted by emitter and received by sensor. By this method, only counts of crossings can be provided, without the possibility of taxonomic determination [8], [9], [19]. However, only few recent studies have compared different monitoring methods on the same wildlife crossing [9], [10], [14], [19]. To our knowledge no study has monitored all three mentioned methods on the same green bridge.

**Figure 1:**
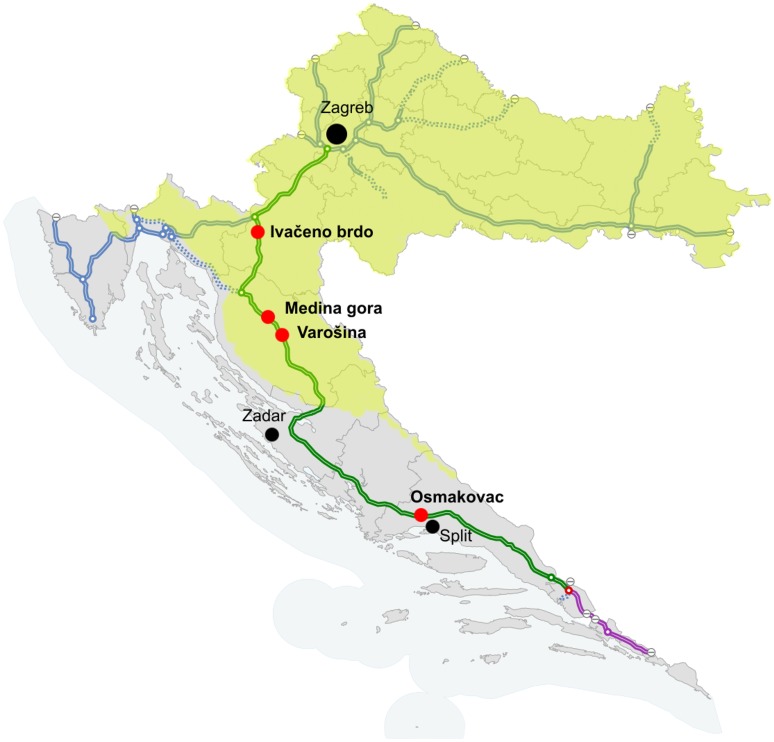
Locations of investigated green bridges along the A1 motorway in Croatia. Climate zones in Croatia are represented as yellow for continental zone, and light blue for Mediterranean zone. Black dots represent the cities. Green bridges along the A1 motorway (green line) are marked as red dots. Map of Croatia representing motorway network was obtained through an Open Access source: http://commons.wikimedia.org. License permissions for the picture can be found at the following link: Autocesta A1, Croatia, highway network, current situation adapted from http://commons.wikimedia.org/wiki/File:Croatia_Autocesta_A1.svg. Attribution: Jeremiah21 [CC BY-SA 3.0 (http://creativecommons.org/licenses/by-sa/3.0/)], via Wikimedia Commons.

On two Croatian motorways data on animal movement for numerous wildlife species has been monitored for the last 14 years. Data on animal diversity and population sizes, adaptation to the presence of the motorway and wildlife crossings across green bridges were collected [8], [20]–[22]. In order to improve overall monitoring of animals on wildlife overpasses, the aim of this study was to use the available dataset obtained for the three year period collected from four green bridges in Croatia (Figure 1) and compare the effectiveness of three different monitoring methods, animal tracks, camera trap monitoring and active IR trail monitoring system. In addition, we have developed an algorithm that dramatically reduces the number of false positive events in the infrared trail dataset and it can be applied to future monitoring.

## Materials and Methods

### Study Area

The A1 motorway (465.5 km) is a major north–south transportation corridor in Croatia (Figure 1). The traffic volume is high with estimated average annual daily traffic of 12,827 vehicles and average summer daily traffic of 29,727 vehicles in 2011 [23]. Both sides of the motorway are fenced with approximately 2 m high wire mesh along entire length. In order to minimize the effects of habitat fragmentation and facilitate migration of animals across the motorway, in total ten green bridges were built and equipped with wildlife monitoring systems. Position of the green bridges along the motorway was determined prior to the present study on the basis of research of bear movements [24] and of animal mortality (mostly bear and wolf) caused by traffic [20], [22], [25].

**Figure 2:**
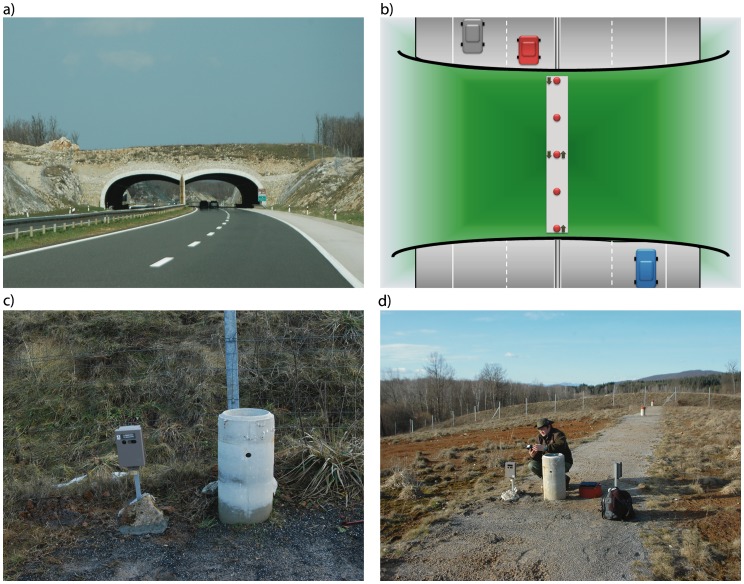
Green bridges overview. (A) Green bridge Medina gora; (B) Location of the track-pad (gray area), concrete tubes with IR sensors (red dots) and camera traps (arrows indicate the direction of cameras) on green bridges; (C) and (D) Camera trap and IR trail setting.

Our study included 335 km of A1 motorway and four green bridges along it: Ivačeno brdo (120 m width, N 45 22.426 E 15 16.161; central part of Croatia), Medina gora (125 m width; N 44 41.916 E 15 23.850; highland part), Varošina (125 m width; N 44 37.842 E 15 26.383; highland part) and Osmakovac (200 m width; N 43 35.291 E 16 26.512; Mediterranean part) (Figures 1, 2). Geographically, three of the bridges – Ivačeno brdo, Medina gora and Varošina are part of the Lika and Gorski Kotar continental climate (average daily temperature during monitoring period was 10.5°C, maximal temperature was 36.9°C, minimal temperature was −22.0°C) and Osmakovac is the part of Mediterranean climate area (average daily temperature during monitoring period was 13.1°C, maximal temperature was 37.1°C, minimal temperature was −16.8°C).

### Field Data Collection

Continuous monitoring of wild animal crossings was executed during the three-year period (1st January 2009 to 30th December 2011). Three indirect monitoring methods were used: digital camera traps, IR detectors and track-pads. Green bridges are purpose-built wildlife crossings and are protected by law as natural values (Nature Protection Act, OG 5/07). Therefore, the permission to perform all planned activities/research/monitoring for the entire defined period was obtained by the competent authority, i.e. Directorate for Nature Protection of the Ministry of Culture of the Republic of Croatia. All data are available upon request.

#### Track-Pad Monitoring

Animal tracks were periodically determined (in average once in 47 days) using track-pads [26], [27] located at the center and spanning the width of the bridge. They were approximately 1.5 m long in the axis of animal movement and covered with 10–15 cm thick layer of track-pad material (Figure 2B, C). Footprints and scats were evaluated and identified to species or species group. The following categories were classified: brown bear (*Ursus arctos*), wild boar (*Sus scrofa*), red deer (*Cervus elaphus*), roe deer (*Capreolus capreolus*), large canids (gray wolf, *Canis lupus* and large dogs, *Canis lupus familiaris*), small canids (red fox, *Vulpes vulpes* and small dogs), badger (*Meles meles*), marten (*Martes* sp.), European hare (*Lepus europaeus*), cattle (*Bos taurus*), human (*Homo sapiens*) and undeterminable tracks. Gray wolf, dog, red fox, badger, marten, European hare and cattle were also determined based on scat. After collecting data, track pads were raked smooth to enable recording of future crossings.

Granulometric analysis of tracking material was performed to enable comparison of tracking substrate among bridges. We have sieved the previously weighed samples through a consecutive set of sieves (mesh size: 4, 2, 1, 0.5, 0.25, 0.125 and 0.063 mm), for 10 minutes each. In this way, eight fractions were yielded and weighed. The percentage of each fraction in the total sample was calculated and, accordingly, the proportion of fine-grained material (silt and clay <0.063 mm), sand (fractions <2 mm and >0.063 mm) and coarse material (debris/gravel >2 mm) was determined for all samples [28].

#### Active Infrared (IR) Trail Monitoring

Active IR systems consist of a set of sensors, an IR transmitter that produces the IR beam and an IR receiver (counter) which registers and records breaking of the beam. In order for an event to be recorded, the animal must break the beam for a specific period of time. The date and time (to the second) is recorded for each event. Four sets of IR transmitters and receivers (TM1550, Trailmaster, Goodson & Associates, Inc. Kansas, USA) were placed on each green bridge, with the exception of Osmakovac with eight sets (due to the width of this bridge, i.e. 200 m). Sets were placed in concrete tubes and were set perpendicular to direction of animal movement so that the distance between the set of IR devices was approximately 25 m (Figures 2B, C). Although this method does not discriminate between species, a partial selection of the animals of interest (medium to large size) was achieved by placing the IR beam at a 0.4 m above the ground and by controlling the time that the beam must be blocked before it registers as an event (-P 10, i.e. 0.5 sec). The data from the IR sensor (Figure 2D) were downloaded using TM Data Collector II (Trailmaster, USA), imported and analyzed by TM StatPack software (Trailmaster, USA).

#### Digital Camera Trap Monitoring

Digital cameras with a passive infrared sensor (PIR) and IR light-emitting-diode flash array equipped with 2 GB compact flash cards (Cuddeback NoFlash, WI, USA) were installed, each covering one segment of the bridge i.e. four per green bridge (Figure 2B). Cameras were in BearSafe (Cuddeback, USA) heavy duty metal cages located close to concrete tubes with IR sensors, roughly 0.5 m off the ground and positioned perpendicular to direction of animal movement (Figure 2C, D). Heat in motion within a detection zone triggered the camera to take 1 photo and 30 seconds of video clip, in order to count the animals that live and move in groups (such as wild boar, roe deer and wolves). Cameras operated continuously throughout the day with delay of 1 minute between two events. The device was configured to have high sensitivity and to automatically adjust its sensitivity level for day and night. IR led light power was set to high (best for distance). Recorded images and videos were analyzed and species and number of individuals were determined. Observed species were: wolves (*Canis lupus*), brown bears (*Ursus arctos*), wild boars (*Sus scrofa*), red deer (*Cervus elaphus*), roe deer (*Capreolus capreolus*), chamois (*Rupicapra rupicapra*), dogs (*Canis lupus familiaris*), red foxes (*Vulpes vulpes*), badgers (*Meles meles*), martens (*Martes* sp.), wild cats (*Felis silvestris*), domestic cats (*Felis catus*), European hedgehogs (*Erinaceus europaeus*), European hares (*Lepus europaeus*), cattle (*Bos taurus*), hooded crows (*Corvus cornix*), common ravens (*Corvus corax)*, common buzzards (*Buteo buteo*), common pheasants (*Phasianus colchicus*), Caspian gulls (*Larus cachinnans*), rock doves (*Columba livia*), pigeons (*Columba* sp.), white wagtails (*Motacilla alba*), blue rock thrush (*Monticola solitarius*), tits (*Parus* sp.) and humans (*Homo sapiens*). Images where species identification was ambiguous were recorded as undeterminable, and those in which there was nothing recorded as blank (no-subject) photos.

### Data Analysis and Presentation

#### IR Data Filtering - Procedure for Reduction of False Positive Infrared Events

**Figure 3:**
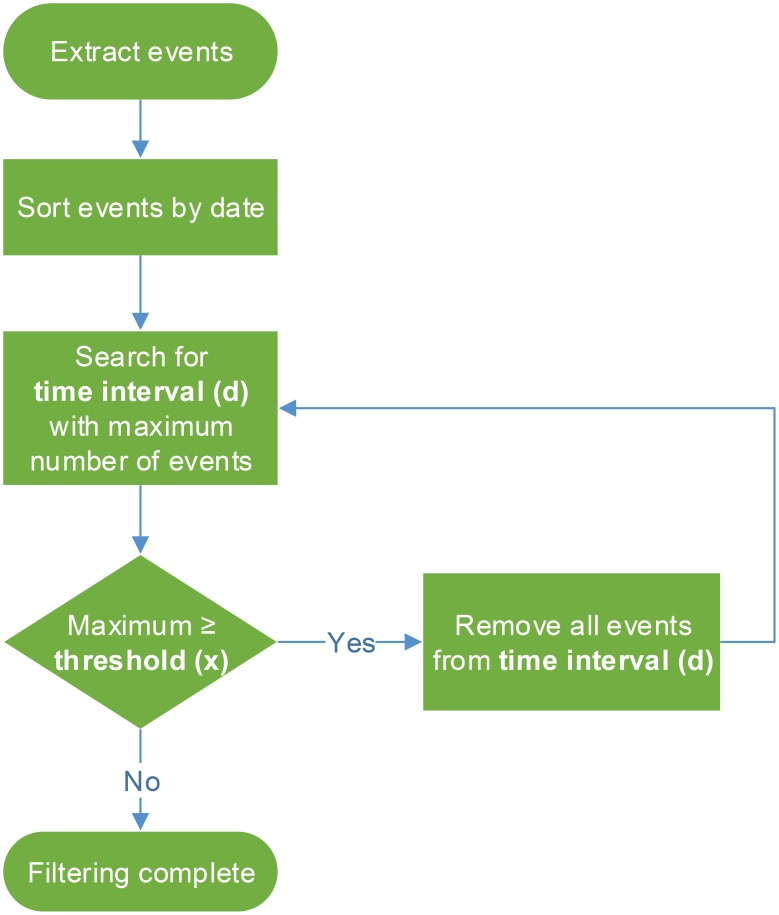
Flow diagram of the IR filtering algorithm.

Preliminary analysis showed high number of events recorded by IR trail system in comparison to events detected by camera traps. Namely, high number of IR events was always recorded during daytime, opposite to camera traps dataset where comparable number of events was recorded during the whole 24-h period. We considered that the camera trap data set reflects natural movement of animals, while repetitive IR events during daytime are probably false positives. Therefore, we have developed a filtering algorithm that searches for repetitive events and removes them from IR dataset (Figure 3). Namely, it uses two user-defined parameters: time interval (*d*) and threshold (*x*). Each IR trailing monitoring unit has its own designator and contains log of events with exact date and time. Data were extracted from the original files and sorted by date. Next, algorithm searches the data set for time interval (*d*) with maximum number of events and if their number is equal or greater than set threshold (*x*) all events from that time frame are removed. Procedure is repeated until there are no more time frames with number of events equal or greater than the *x*, and then filtering is completed.

#### Statistical Analysis

Species composition on different wildlife bridges as determined by two methods - track pads and camera traps was presented as percentages and the data was analyzed with the Chi-squared test for the comparison of two proportions (from independent samples). Chi-squared test was performed with MedCalc software, a complete statistical program for Windows (Version 13.2.2, MedCalc Software, Ostend, Belgium). For the comparison of IR data before and after filtering we used Mann-Whitney U test, implemented in the STATISTICA 7 software (StatSoft, Inc., USA).

## Results

### Overview of Total Number of Crossings

Initial analysis of the total number of crossing events during 3-year monitoring period for all four bridges is presented in Table 1. Based on the track-pad and camera trap monitoring, highest total number of events was recorded at green bridge Ivačeno brdo, followed by Osmakovac, Varošina and finally Medina gora. When IR monitoring system was used, highest total number of events was recorded at green bridge Osmakovac, followed by Ivačeno brdo, Varošina and Medina gora. Presumably, animal crossings recorded by camera for Osmakovac were underestimated. This green bridge is app. two times wider than other three bridges, and, although the same protocol was used, its width was not completely covered by cameras.

**Table 1:** Number of crossing events recorded by different monitoring methods.

Green bridge	Monitoring by tracks				Camera trap monitoring				Infrared trail monitoring			
	2009	2010	2011	Total	2009	2010	2011	Total	2009	2010	2011	Total
Ivačeno brdo	327	155	314	796	1,798	1,859	1,625	5,282	23,158	19,035	16,521	59,453
Medina gora	191	127	148	466	307	615	732	1,654	5,907	10,441	10,879	27,227
Varošina	179	147	189	515	1,267	847	861	2,975	23,316	16,110	13,619	53,035
Osmakovac	326	127	198	651	2,229	1,186	1,550	4,965	26,585	37,189	32,769	96,543

Numbers of events per year and per green bridge are given.

Further, for individual bridges, IR detectors consistently gave the highest total number of recorded events, followed by camera traps, and track-pads which gave the lowest number of events (Table 1). As an example, on the bridge Ivačeno brdo we recorded 59,453 by IR detectors sensors, 5,282 by camera and 796 events by track-pads.

### Species Composition Determination by Camera Trap and Track-Pad Monitoring

**Figure 4:**
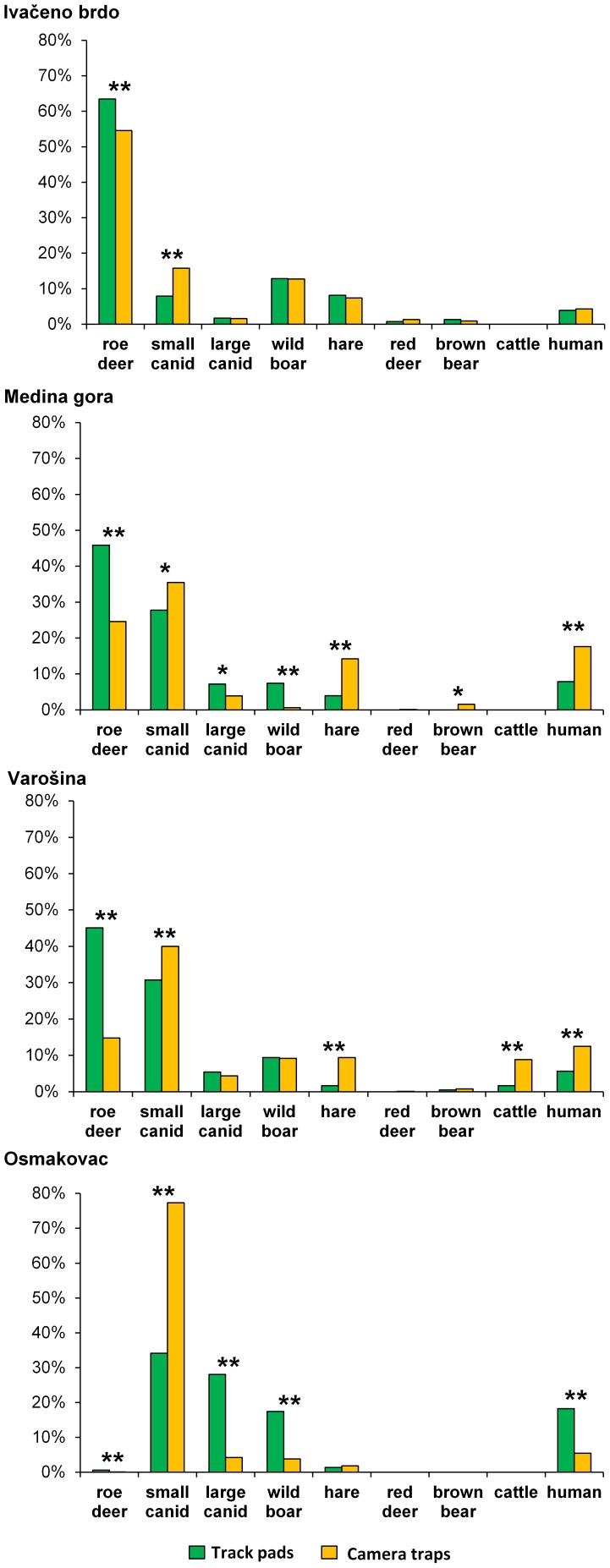
Species composition on green bridges. Small canid category included fox and small dog and large canid category included large dog and wolf. Undetermined tracks, empty photographs and animal species that occurred sporadically on green bridges (e.g. marten, wild cat) were excluded from the analysis. Asterisks indicate statistically significant difference for the comparison of two proportions (Chi-square test; *p<0.05; **p<0.001).

Species composition analysis by track pads and cameras revealed the highest abundance of roe deer and small canid categories on green bridges Ivačeno brdo, Medina gora and Varošina, located on the continental part of A1 motorway (Figure 4). On Mediterranean part of A1 motorway, where green bridge Osmakovac is located, roe deer, red deer and brown bear are scarce and thus extremely rarely detected by both methods. However, on this green bridge small canid category is abundant (Figure 4). On all four green bridges both methods recorded human activity (Figure 4).

Via track-pads we obtained different percentages of undetermined tracks on different green bridges: 16.1% on Ivačeno brdo, 22.3% on Medina gora, 28.3% on Varošina, and 35.4% on Osmakovac. This result led us to suspect that there might be differences in the quality of track pad material and the performed granulometric analysis confirmed this assumption (Figures S1, S2). On all four green bridges the most represented particles were from fractions 2–4, 1–2 and 0.5–1 mm (≥15% each), while the particles from the largest fraction (>4 mm) as well as the ≤0.5 mm fractions were less represented (3 to 13%), as seen in Figure S1. Further, the proportion of fine-grained material (silt and clay) was highest on the track-pad samples taken on the green bridges Ivačeno brdo and Varošina (8 and 9%, respectively), while other two green bridges displayed less silt and clay (4% on Osmakovac and 5% on Medina gora; see Figure S2). Further, Osmakovac exhibited higher proportion of sand (75%) in comparison to Ivačeno brdo, Varošina and Medina gora (60–64%).

Next, we determined species composition on different green bridges and found statistically significant differences between percentages of particular animal species (Figure 4 and Table S1). For some green bridges, both methods showed significantly different percentages for almost all categories of animals (e.g. Osmakovac and Medina gora). In contrast, when examining the data for Ivačeno brdo, both methods gave comparable results for large canids, wild boar, European hare, red deer, brown bear, badger and human categories. However, recorded percentages of roe deer and small canid categories were statistically different, depending on the method used (Chi-square test, *p*<0,001).

Species sporadically detected by cameras across all four green bridges were not included in the analysis shown in Figure 4. They were: chamois (*Rupicapra rupicapra*), badger (*Meles meles*), marten (*Martes* sp.), wild cat (*Felis silvestris*), domestic cat (*Felis catus*), European hedgehog (*Erinaceus europaeus*), hooded crow (*Corvus cornix*), common raven (*Corvus corax)*, common buzzard (*Buteo buteo*), common pheasant (*Phasianus colchicus*), Caspian gull (*Larus cachinnans*), rock dove (*Columba livia*), pigeon (*Columba* sp.), white wagtail (*Motacilla alba*), blue rock thrush (*Monticola solitarius*) and tits (*Parus* sp.).

### Active IR Data Analysis

Initial analysis showed high number of events recorded by IR trail system in comparison to camera traps (Table 1). To analyze the causes of this discrepancy, we have checked the number of IR events for: (i) every recorded photograph or video clip (No of IR monitoring events/No of photographs, blank photograph events included); (ii) every photographed animal (No of IR monitoring events/No of individuals) (Table 2). This analysis showed from 0.43 to 1.05 IR events per photograph and from 0.51 to 1.29 events per individual animal. The fact that these ratios are mostly <1 indicates that high total number of IR events is not primarily caused by events that correspond to camera monitoring results.

**Table 2:** IR trail *vs.* camera traps - recording of the same crossing event.

Green bridge	No of IR events/No of photos	No of IR events/No of individuals
Ivačeno brdo	0.94	0.75
Medina gora	0.59	0.82
Varošina	1.05	1.29
Osmakovac	0.43	0.51

No of IR events/No of photos  =  ratio of the number (No) of IR monitoring events and the number (No) of photos taken at the same time point (±5 min). Photos events include all animals and humans, undetermined and blank images.

No of IR events/No of individuals  =  ratio of the number (No) of IR monitoring events and the number (No) of photographed individuals taken at the same time point (±5 min).

**Figure 5:**
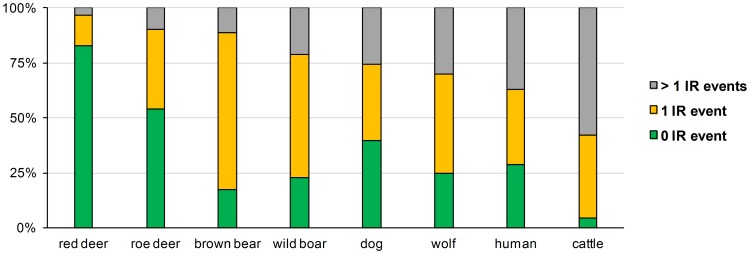
Number of IR events (0, 1, >1) per photographed large animal/human.

Next, we looked at the number of IR events triggered by individual large animal crossings, as represented in Figure 5. Certain species commonly make multiple IR events per photographed individual (e.g. cattle; >55%). In contrast, fast moving animals like red deer and roe deer are often not recorded (>50%) by IR trail monitoring system. Third category are species such as brown bear and wild boar which most often (>55%) make 1 IR event per photograph.

**Figure 6:**
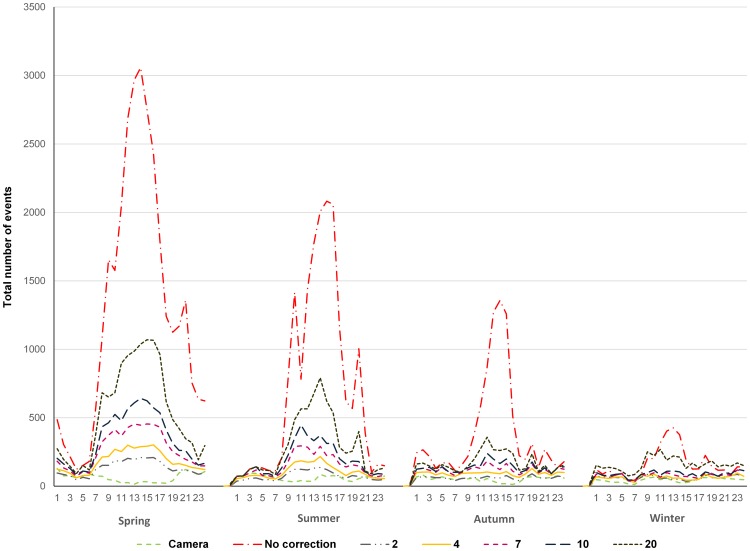
Total number of recorded events on the green bridge Ivačeno brdo. All recorded events during the three-year monitoring period, per hour of the day, within each season (spring, summer, autumn, winter) were totaled. Camera - total number of events recorded by camera traps; No correction - total number of events recorded by IR trail monitoring; 2, 4, 7, 10, 20 - number of IR events after filtering using different threshold values (*x*), respectively.

Majority of IR events did not correspond to camera dataset and we hypothesized that they are false positives. To further analyze this, we have divided the total IR events per season (spring, summer, autumn, winter) and within each season IR events were summed per hour of the day. Throughout the three-year monitoring period a similar pattern emerged on all four green bridges (Figures 6 - no correction, Figure S3). Namely, a peak of IR events was always present during daytime (from app. 9:00 AM to app. 6:00 PM). When we analyzed data from camera traps in the same manner, this peak was absent. Camera data set showed comparable number of recorded events during the whole 24-h period, irrespective of the analyzed season and green bridge, i.e. approximately flat-shaped curve (Figures 6 - Camera, S3). Also, IR events recorded during daytime often showed patterns not characteristic for wild animal crossings (e.g. high number of repetitive events during several hours) and caused the observed peak in the curve. Therefore, we considered these repetitive events as false positives and developed an algorithm for their reduction (Figure 3). The aim was to flatten the IR data curve, as flat-shaped curve is more representative for animal movements according to camera data set. Filtering algorithm searches for repetitive events and removes them from IR dataset, using two user-defined parameters: time interval (*d*) and threshold (*x*) (see Materials and Methods section and Figure 3 for details). We defined time interval *d* to 60 minutes and regarding threshold, we tested several different *x* values (2, 4, 7, 10 and 20). As can be seen in Figure 6, flattening of IR curves was more pronounced when smaller *x* values were applied. This was consistent for all green bridges and all seasons. All curves were statistically significantly different (Mann-Whitney U test, *p*<<0.0001). In conclusion, we preferred threshold value *x* = 4 which resulted in removal from 74.84 to 89.05% of IR events (Table 3).

**Table 3:** Removal of IR events by filtering.

Green bridge	No filtering	Threshold 4	Removed events (%)
Ivačeno brdo	59,453	10,803	81.83
Medina gora	27,227	6,850	74.84
Varošina	53,035	8,631	83.73
Osmakovac	96,543	10,575	89.05

Total number of IR events before filtering (No filtering) and after filtering (i.e. when a time interval of 60 minutes contained more than four events, all events from that time interval were removed  =  Threshold 4).

## Discussion

In presented study, total number of crossing events and species composition patterns were different on each of four monitored bridges (Table 1, Figure 4). This result was expected and reflected multiple factors that can affect the number of various animal crossings in space and time; e.g. composition of fauna, population densities, position of the bridge with the respect to movement corridors, quality of landscape architecture of the bridge and other technical characteristics, such as traffic noise and light protection etc. [10], [12], [29]. However, the analysis of these differences was not in the scope of this work, as we aimed to compare the methods in terms of their ability (1) to detect different wildlife species, and (2) to give good estimation of number of animal crossings.

Regarding (1), the ability of the methods to detect different wildlife species, we compared the results of track-pad and camera trap monitoring and found statistically significant differences in detection of certain animal species on some green bridges (Figure 4). One of the factors that could cause this discrepancy is the low quality of track pad material, causing the underestimation of some species by track pad monitoring and increasing the number of undeterminable tracks. Granulometric analysis showed correlation of the quality of track pad material and accuracy of track determination, i.e. low percentage of fine-grained material (i.e. silt and clay) and high percentage of sand correlated with high proportion of undetermined tracks (see Results and Figures 4, S1, S2). Also, Osmakovac track-pad material had highest quantity of 1–2 mm particles, and we hypothesize that this fraction could fill in the tracks, making them undeterminable. Similar granulometric analyses of track pad material are rare in the literature. Mixture of sand and silt was recommended as a tracking material for detection of medium and large sized mammals, and soothed track plates for small and medium sized [14]. Marble dust was used to detect a variety of animals, from frogs and lizards to large canids [16], [30]. For detection of birds *Rhinoptilus bitorquatus* track-pad material consisting of particles smaller than 1 mm (50% smaller than 27 µm) was reported [31].

Next, we compared the results of track-pad and camera trap monitoring (Figure 4) and found that the tracks of small canids were underestimated in comparison to proportion obtained by camera traps. This could be explained by the track-pad material with high proportion of sand that is more suitable for larger animals [10], [14]. Therefore, we concluded that monitoring by camera was in our case preferable method for small canids detection. Similar results were reported previously [32]. On the contrary, Ford *et al.* showed that small canids detections were 4 times more likely to occur by track-pad than camera [10]. However, these track-pads were positioned in the underpasses, while in our case track-pads were uncovered and affected by weather conditions.

Second example of statistically significant incongruence between species representation by track pads and by camera traps is roe deer category. Continuously higher ratio of roe deer was obtained with tracks, concluding that their number was underestimated by camera trap monitoring. These large animals leave deep, long-lasting, easily detectable tracks, even on the track pads with poor quality tracking material. The greater detection rate of roe deer by track-pads could be explained by their fast traveling speed, which may exceed the sensitivities of the motion sensor on the camera. Previous studies are indicating that camera trap method has numerous advantages including more reliable species identification [10], [14], [33]. However, camera should not be taken as the best possible approach for estimating the population size of certain species, e.g. roe deer. Further, it is clear that camera, as well as other methods used here, were not designed for smaller/lower-to-the-ground species as they were detected only sporadically. Their detection could be improved with lowering of IR beam of the trailmaster (below 40 cm) or with better composition of track pad material. Nevertheless, cameras are the best and most widely used method for detection of small mammals [17], [34]–[36]. Regarding (2), the ability of the method to give good estimation of number of animal crossings, an ideal situation would be that each individual animal crossing is recorded as one event. However, we observed a striking difference in total number of events recorded by different methods, i.e. infrared trail monitoring system gave from 11 to 19 times more events than camera traps, as reported previously [8], [37]. In contrast, track-pads gave the lowest total number of events, but this was to expect because we did not use track pad method for accurate assessment of absolute number of crossings, but for evaluating species composition on the green bridges. In order to accurately determine the number of events by track-pads, they need to be checked frequently (e.g. every second day), but this implies very high long-term costs [10], [38]. However, to analyze species composition we presumed that high frequency of visits was irrelevant, and we recorded tracks in average once in 47 days. Therefore, the low number of total events recorded by track-pads is understandable.

Discrepancy between the number of events recorded by camera traps and IR trail system can be caused by any of the following: (i) camera traps or IR trail system do not record all crossings (increasing the number of false negative events); (ii) individual crossing event (i.e. the same animal) can activate IR trail system multiple times (increasing the number of false positive events) because of slow movement and/or animal residing for prolonged time on the bridges; (iii) camera traps or IR trail system can be activated by other factors, and not only by real crossing events (increasing the number of false positive events).

Although we cannot state that camera trap monitoring detects all crossings (see below for more details), each detected event (i.e. photograph) corresponds to a real crossing event (true positive). In contrast, for IR trail events we cannot discriminate real crossing events (true positives) from false positives. Therefore, we took photographs as a base for our analysis. We calculated the average number of IR events per photograph or per photographed individual and, unexpectedly, this ratio was below 1 for all green bridges except Varošina (Table 2). Therefore, we can conclude that, although overall data show high number of IR events in comparison to other methods, IR trail system does not record all crossings. This is in particular true for small animals (hare, badger, fox, marten etc.), because their height is below the IR beam (0.4 m). Detection of some animal species is more accurate than others, which depends on the size, speed of movement and length of time spent on the green bridges. For some animal species (i.e. cattle) one individual usually activates IR trail system multiple times, for others there is less than 1 IR event per photographed animal (i.e. roe deer). Finally, the brown bear presents an exception, as it comes close to the ideal situation of one animal crossing that is recorded as one event by both IR trail and camera traps (Figure 5). When comparing these results with species composition on different green bridges we observed interesting correlation. Namely, presence of cattle (app. 10%) on Varošina corresponded with unusually high No of IR/No of individuals ratio (1.29; Table 2) for this bridge. To our knowledge, this is the first time that correlation of IR data *vs*. photographs taken at the same time point was analyzed.

When analyzing the number of IR events per season and hour in the day, we observed a strong peak of IR events during daytime, that was most pronounced in the spring. Further, as mentioned previously, we considered the camera traps being the most accurate (although by no means perfect) method, and for this data the same peak was absent i.e. comparable number of events were recorded during the whole 24-h period. We have further manually inspected the IR datasets and observed temporally clumped events during prolonged time (i.e. several hours) usually during daytime, therefore corresponding to the observed peak. They cannot be ascribed to animal activity, and were most probably caused by other factors such as high vegetation moved by wind, heavy snowfall and rainfall, rapid changes in sunlight (reflectivity), heat and other external conditions, e.g. wasps and lizards that used the concrete tubes as nests and entered using holes in front of IR device [9], [37], [39]–[42]. Additional previously reported reasons for false triggering were unstable mounting of the IR device and oversensitivity (5 pulses) [19], [39], [41], but in our case these can be excluded because the IR equipment was stably fixed in concrete tubes (Figure 2C) and sensitivity was set to 10 pulses (0.5 sec). Peak of IR data observed here corresponds well with the peak of surface wind speed in the early afternoon over most of the globe [43]. Further, the fact that the peak is strongest in the spring corresponds to growing of vegetation and small animal activity that are most pronounced in this season. Some of the proposed causes for false positive IR events could be prevented (e.g. removal of vegetation), but others are unavoidable (e.g. wind, heat, heavy rainfall).

We have to point out that camera trap monitoring, despite the approximately realistic curve of seasonal animal crossings, cannot detect all crossings due to its technical limits. Camera traps used here were triggered by Passive Infrared (PIR) detectors that are motion/heat sensors used for detection of heat radiated from the body of an animal moving across the field of view. They exhibit optimal performance when there is a large difference between the air temperature and the animal, while small difference results in a greatly reduced detection range. Higher air temperatures are generally associated with higher error rates. Also, large animals are easier to detect than small ones, i.e. the PIR sensor will detect small animals at close range, but may miss them at farther distance [19], [36], [37], [44]–[46]. Small variations in camera orientation can significantly influence the results [36], [45]. Further, there was a minimum 1 minute delay between two events recorded by camera, and during this time new animal crossings could not be detected (e.g. wild boar flock). Overall, we assume that total number of real events is *higher* than total number of camera events, but approximately follows the shape of the curve obtained from camera records for 24 hour period.

We developed a filtering algorithm for the reduction of false positive IR events, thus offering automated approach that can be easily adapted to novel situations. Out of different tested threshold values, threshold value *x* = 4 was chosen as the most realistic one because it produced approximately flattened 24 hour curve, slightly higher than the curve produced from camera dataset. It is possible that, on rare occasions, four or more animals crossed the same section of bridge in the same interval of sixty minutes, but we wanted to make sure not to overestimate the use of the bridge. Threshold value of *x* = 2 was discarded because during fall and winter the IR curve corrected by this value were below corresponding camera curves (Figure 6). We also regarded this value as unrealistic because often 2 animals crossed the bridge in the same hour. High number of false positive events obtained by IR trail monitoring system was noticed previously but another reduction approach was used [8]. The mean values and standard deviations of the number of records per hour per section of the bridge were calculated and hours that had seven or more records (more than one SD above the mean) were filtered out. However, we obtained better results when we used the threshold value *x* = 4 (Figure 6).

## Conclusions

We have attentively analyzed and compared the effectiveness of three different monitoring methods, animal tracks, camera trap monitoring and active infrared trail monitoring system. In overall, all methods have their advantages and disadvantages, as it is summarized in Table 4. Although expensive in the long-term, track-pad monitoring can be used to determine species composition, even if track-pads are not visited frequently. Nevertheless, the quality of the track pad material has to be appropriate, i.e. higher proportion of fine-grained material is better, as shown by our granulometric analysis. Camera monitoring are reliable for species identification, exhibit most affordable long-term operating costs, and are less sensitive to weather conditions and level of animal activity [10], [14], [33]. However, cameras underestimated the number of fast moving roe deer that were very abundant on some of the tested wildlife bridges. This could be overcome by comparison with track-pad data, since roe deer leave long lasting and deep tracks. Further, cameras underestimate the total number of animal crossings [36], [45]. Finally, IR trail system cannot give information on species composition and greatly overestimates the total number of animal crossing events. Therefore, we have developed a method for reduction of false positive events from IR dataset, and removed IR events that were presumably caused by other factors, and not by animal crossings. In our case, overall around 80% of total IR events were removed, but the method should be calibrated when used in other setting. After filtering, we used IR monitoring to approximate the real number of crossings, which could not be done using the camera dataset. In conclusion, combination of different methods should be chosen depending on the aims of the particular monitoring study (Table 4).

**Table 4:** Comparison of different monitoring methods.

Purpose		Monitoring by tracks	Camera trap monitoring	Infrared trail monitoring
**Species identification**	Reliability	Reliable, but depends on the track pad material and condition	Highly reliable, but can miss fast moving animals (e.g. roe deer)	
				Not possible
	Improvements	Use higher proportion of fine-grained material	Combine with track pads for better species coverage	
**Wildlife crossings estimation**	Estimation bias	Underestimated	Underestimated	Overestimated
	Improvements	Frequent field visits (expensive!) or combination with other methods	Combination with other methods	Usage of filtering algorithm tuned by camera trap data to improve wildlife crossing estimation

## Supporting Information

Figure S1 **Different particle size fractions of the track-pad material.**
(TIF)

Figure S2 **Granulometric composition of the track-pad material.**
(TIF)

Figure S3 **Total number of recorded events on the green bridges.** (A) Medina gora, (B) Varošina and (C) Osmakovac. All recorded events during the three-year monitoring period, per hour of the day, within each season (spring, summer, autumn, winter) were totaled. Camera - total number of events recorded by camera traps; No correction - total number of events recorded by IR trail monitoring; 4 - number of IR events after filtering using threshold value (*x* = 4).
(TIF)

Table S1 **Additional information for the Chi-square test for the comparison of two proportions presented in** **Figure 4** **.**
(DOCX)
