# Supplementary figures and images for: Comparative Analysis of Three Different Methods for Monitoring the Use of Green Bridges by Wildlife
Source: PLoS One. 2014 Aug 29;9(8):e106194. doi: 10.1371/journal.pone.0106194 (PMC4149566; doi:10.1371/journal.pone.0106194)

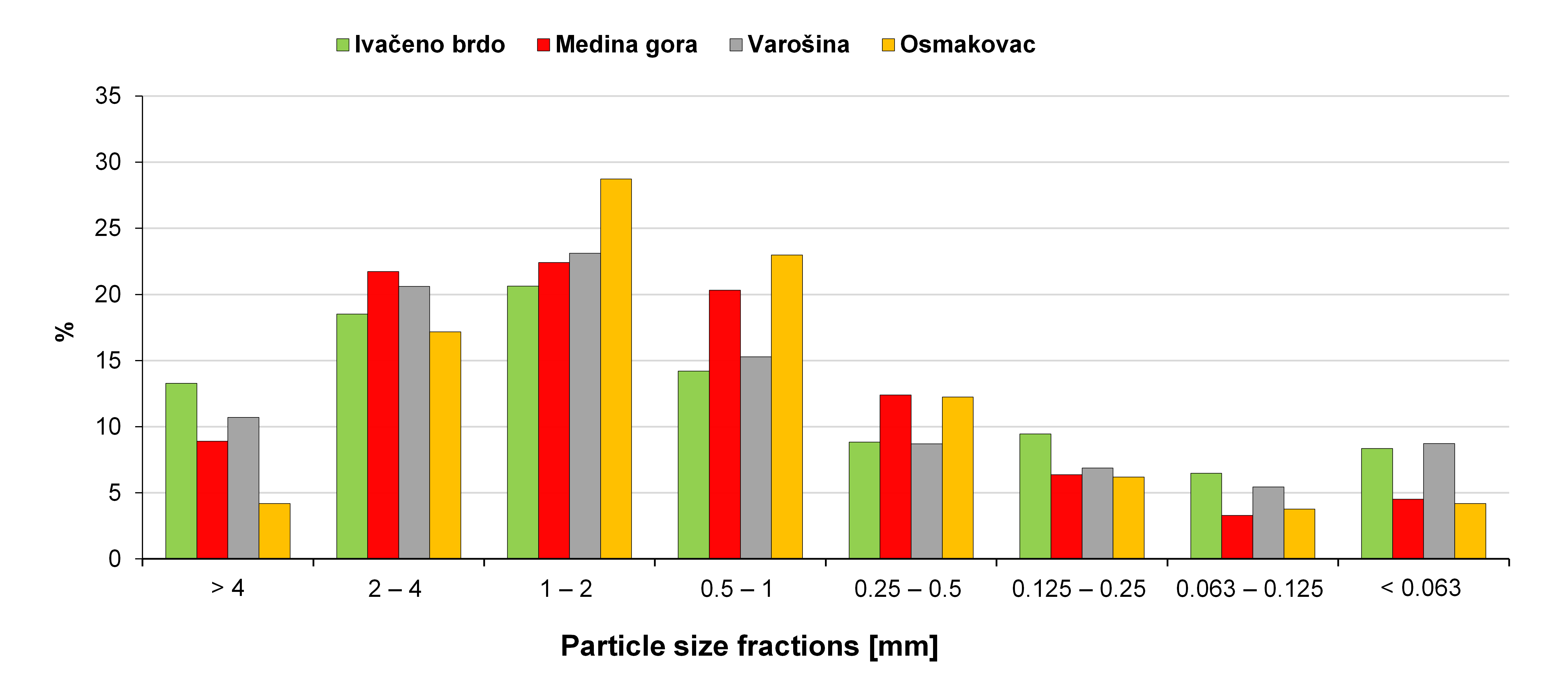

Supplement: Figure S1 — Different particle size fractions of the track-pad material. (TIF) [file pone.0106194.s001.tif]

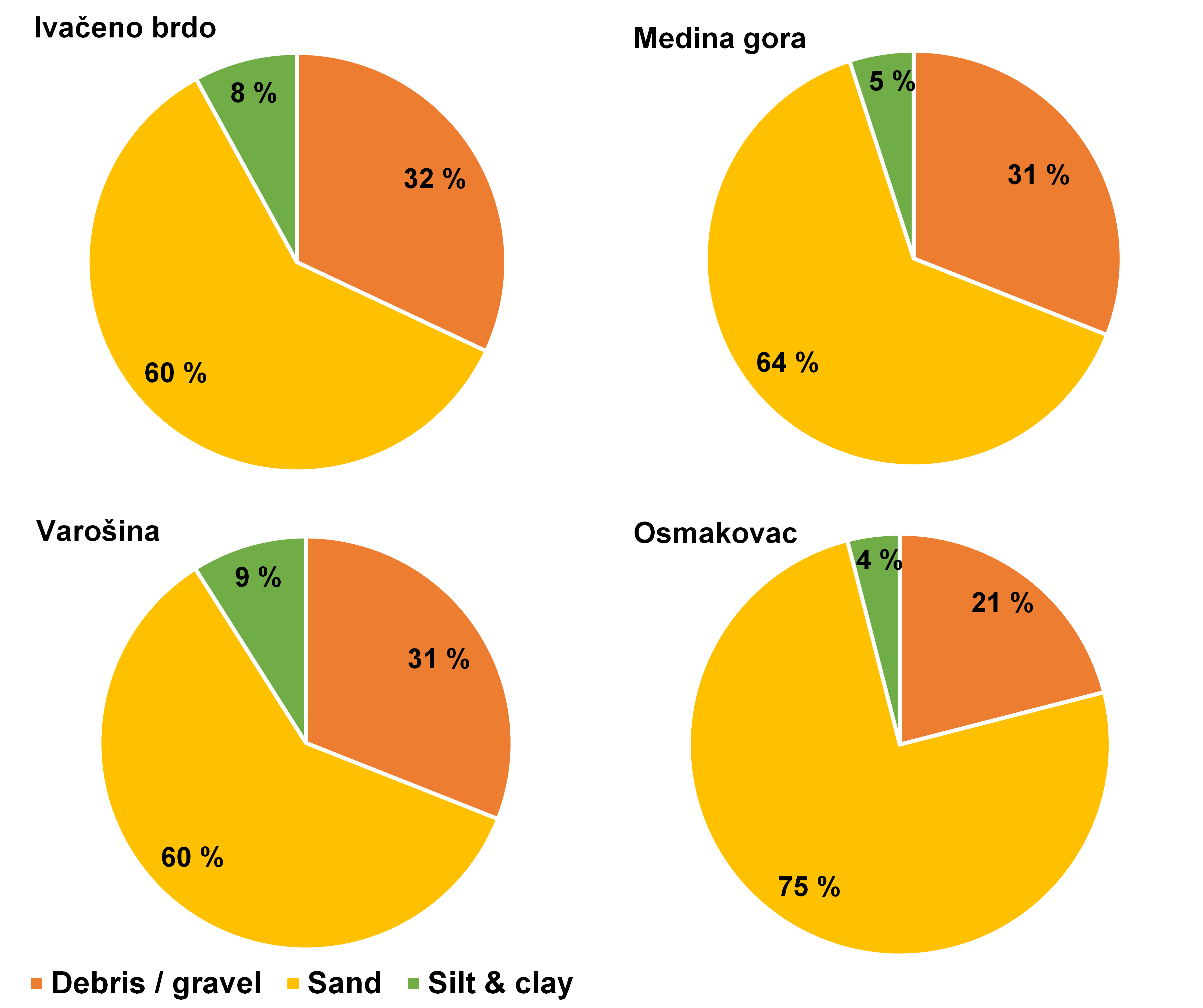

Supplement: Figure S2 — Granulometric composition of the track-pad material. (TIF) [file pone.0106194.s002.tif]

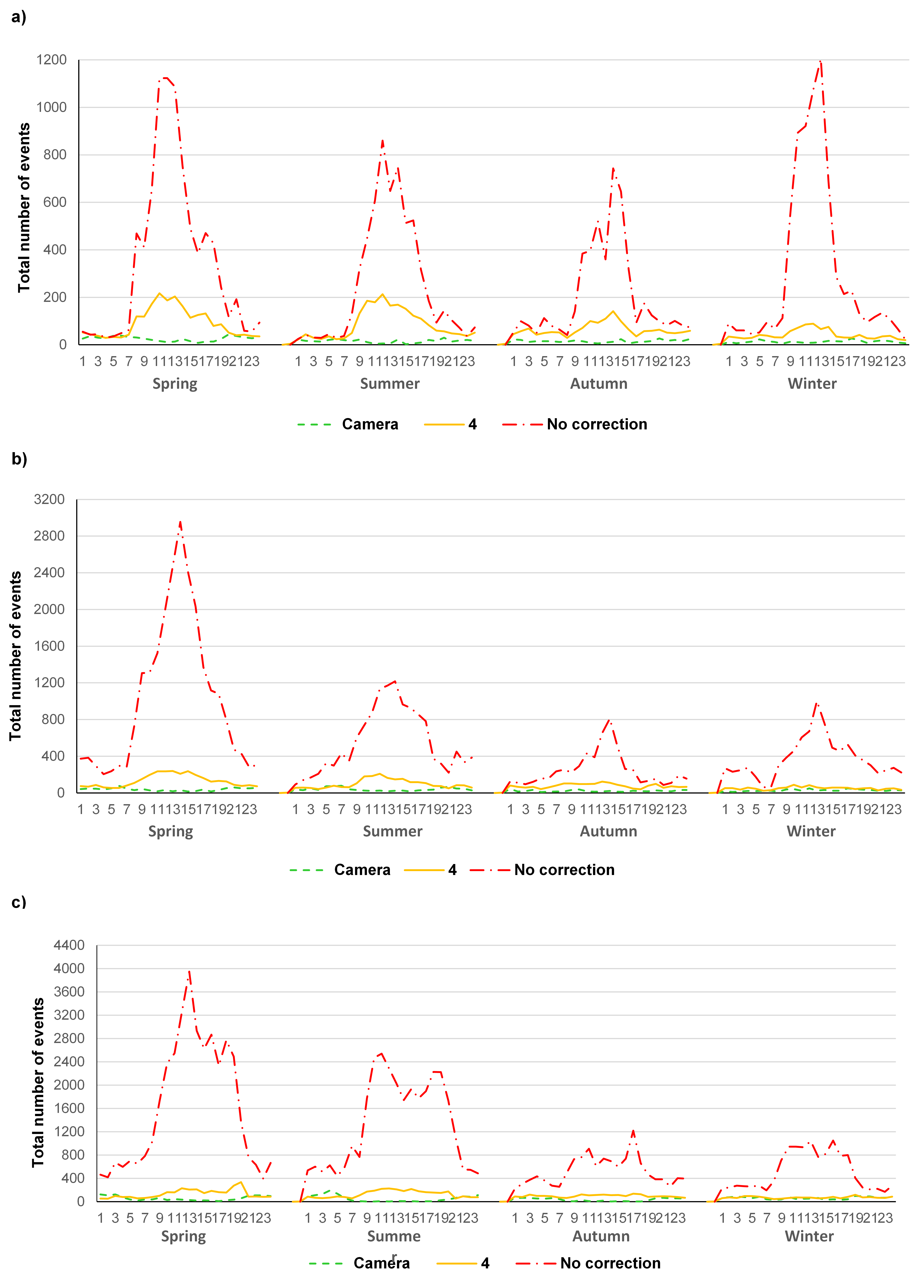

Supplement: Figure S3 — Total number of recorded events on the green bridges. (A) Medina gora, (B) Varošina and (C) Osmakovac. All recorded events during the three-year monitoring period, per hour of the day, within each season (spring, summer, autumn, winter) were totaled. Camera - total number of events recorded by camera traps; No correction - total number of events recorded by IR trail monitoring; 4 - number of IR events after filtering using threshold value (x = 4). (TIF) [file pone.0106194.s003.tif]
