# Supplementary material for: Comparative Analysis of Three Different Methods for Monitoring the Use of Green Bridges by Wildlife
Source: PLoS One. 2014 Aug 29;9(8):e106194. doi: 10.1371/journal.pone.0106194 (PMC4149566; doi:10.1371/journal.pone.0106194)
Supplement: Table S1 — Additional information for the Chi-square test for the comparison of two proportions presented in Figure 4 . (DOCX) [file pone.0106194.s004.docx]

**Table S1. Additional information for the Chi-square test for the comparison of two proportions presented in Figure 4.**

| **Green bridge** | **Comparison of proportions** | **Roe deer** | **Small canid** | **Large canid** | **Wild boar** | **European hare** | **Red deer** | **Brown bear** | **Cattle** | **Human** |
| --- | --- | --- | --- | --- | --- | --- | --- | --- | --- | --- |
| **Ivačeno brdo** | **Camera sample size** | 3556 | 3556 | 3556 | 3556 | 3556 | 3556 | 3556 | 3556 | 3556 |
|  | **Camera percentage (%)** | 55 | 16 | 2 | 13 | 7 | 1 | 1 | 0 | 4 |
|  | **Tracks sample size** | 824 | 824 | 824 | 824 | 824 | 824 | 824 | 824 | 824 |
|  | **Tracks percentage (%)** | 63 | 8 | 2 | 13 | 8 | 1 | 1 | 0 | 4 |
|  | **Chi-square** | 17.097 | 33.903 | 0.0191 | 0.0033 | 0.858 | 0.0377 | 0.0377 | NA | 0.00973 |
|  | **Degrees of freedom** | 1 | 1 | 1 | 1 | 1 | 1 | 1 | NA | 1 |
|  | **Significance level (P)** | **< 0.0001** | **< 0.0001** | 0.8902 | 0.9542 | 0.3542 | 0.846 | 0.846 | NA | 0.9214 |
| **Medina gora** | **Camera sample size** | 958 | 958 | 958 | 958 | 958 | 958 | 958 | 958 | 958 |
|  | **Camera percentage (%)** | 25 | 35 | 4 | 1 | 14 | 0 | 2 | 0 | 18 |
|  | **Tracks sample size** | 432 | 432 | 432 | 432 | 432 | 432 | 432 | 432 | 432 |
|  | **Tracks percentage (%)** | 46 | 28 | 7 | 7 | 4 | 0 | 0 | 0 | 8 |
|  | **Chi-square** | 59.855 | 6.303 | 5.093 | 36.393 | 29.655 | NA | 7.351 | NA | 22.709 |
|  | **Degrees of freedom** | 1 | 1 | 1 | 1 | 1 | NA | 1 | NA | 1 |
|  | **Significance level (P)** | **< 0.0001** | **0.0121** | **0.024** | **< 0.0001** | **< 0.0001** | NA | **0.0067** | NA | **< 0.0001** |
| **Varošina** | **Camera sample size** | 1972 | 1972 | 1972 | 1972 | 1972 | 1972 | 1972 | 1972 | 1972 |
|  | **Camera percentage (%)** | 15 | 40 | 4 | 9 | 9 | 0 | 1 | 9 | 12 |
|  | **Tracks sample size** | 426 | 426 | 426 | 426 | 426 | 426 | 426 | 426 | 426 |
|  | **Tracks percentage (%)** | 45 | 31 | 5 | 9 | 2 | 0 | 0 | 2 | 6 |
|  | **Chi-square** | 192.817 | 11.619 | 0.643 | 0.00871 | 23.023 | NA | 3.157 | 23.023 | 12.341 |
|  | **Degrees of freedom** | 1 | 1 | 1 | 1 | 1 | NA | 1 | 1 | 1 |
|  | **Significance level (P)** | **< 0.0001** | **0.0007** | 0.4226 | 0.9256 | **< 0.0001** | NA | 0.0756 | **< 0.0001** | **0.0004** |
| **Osmakovac** | **Camera sample size** | 3303 | 3303 | 3303 | 3303 | 3303 | 3303 | 3303 | 3303 | 3303 |
|  | **Camera percentage (%)** | 0 | 77 | 4 | 4 | 2 | 0 | 0 | 0 | 5 |
|  | **Tracks sample size** | 509 | 509 | 509 | 509 | 509 | 509 | 509 | 509 | 509 |
|  | **Tracks percentage (%)** | 1 | 34 | 28 | 17 | 1 | 0 | 0 | 0 | 18 |
|  | **Chi-square** | 26 | 396.068 | 376.397 | 135.459 | 1.893 | NA | NA | NA | 116.586 |
|  | **Degrees of freedom** | 1 | 1 | 1 | 1 | 1 | NA | NA | NA | 1 |
|  | **Significance level (P)** | **< 0.0001** | **< 0.0001** | **< 0.0001** | **< 0.0001** | 0.1689 | NA | NA | NA | **< 0.0001** |

**P < 0.05**

NA - not applicable
